# Systematic evaluation of neuro-ophthalmic outcomes of the ophthalmic artery covered by flow-diverter

**DOI:** 10.3389/fneur.2025.1479229

**Published:** 2025-03-18

**Authors:** Yu-Hu Ma, Yong-Lin He, Sen Lin, Ya-Wen Pan, Chang-Wei Zhang

**Affiliations:** ^1^Department of Neurosurgery, West China Hospital, Sichuan University, Chengdu, Sichuan, China; ^2^Department of Neurosurgery, The Second Hospital of Lanzhou University, Lanzhou, China

**Keywords:** flow-diverter, carotid-ophthalmic aneurysms, ophthalmic artery, neuro-ophthalmic complications, systematic review

## Abstract

Carotid-ophthalmic aneurysms (COA) are complex and severe intracranial arterial lesions, and their treatment and management have always been a focus of clinical research. In recent years, the introduction of flow diverters (FD) has provided a revolutionary method for the treatment of intracranial aneurysms (IA). Although FD has achieved significant success in reducing the risk of COA rupture, the complex anatomical structure and critical function of the ophthalmic artery (OphA) mean that covering the OphA with FD may lead to adverse ophthalmic outcomes. This review aims to systematically examine the ocular complications and their mechanisms when FD covers the OphA in the treatment of COA, emphasizing the potential risks that clinicians should be aware of when applying FD treatment, to reduce complications and improve the overall prognosis of patients.

## 1 Introduction

**Figure 1:**
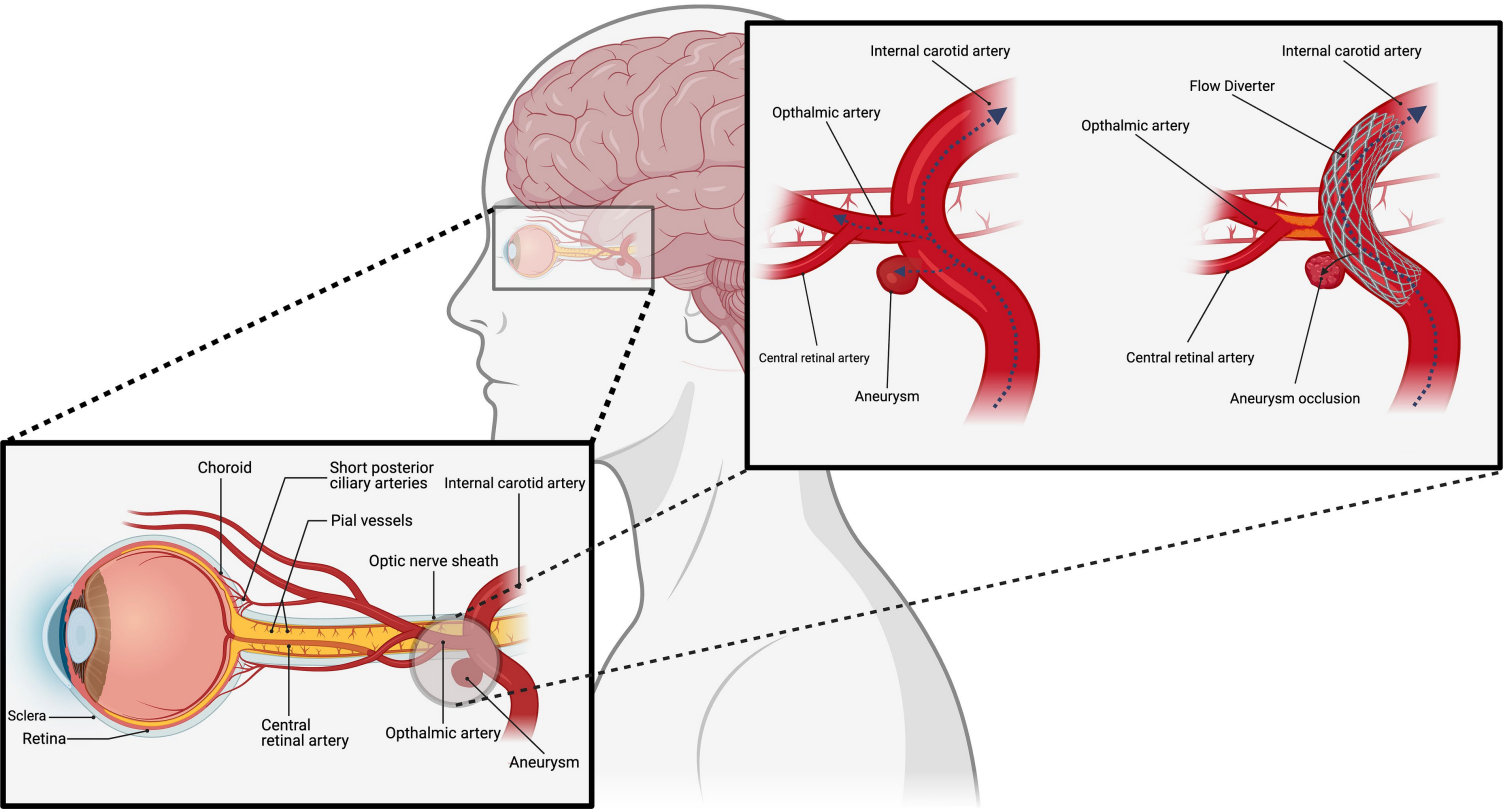
Flow diverters for the treatment of carotid-ophthalmic aneurysms.

Carotid-ophthalmic aneurysms (COA) represent a significant intracranial arterial condition, and their treatment and management have long been central to clinical research (1). The advent of flow diverters (FD) in recent years has revolutionized the treatment of intracranial aneurysms (IA) by altering hemodynamics to promote aneurysm occlusion (Figure 1) (2). While FDs have demonstrated remarkable success in mitigating the risk of aneurysm rupture, the potential ocular complications associated with covering the ophthalmic artery (OphA) during their use warrant careful consideration.

As the primary branch of the internal carotid artery (ICA), responsible for supplying blood to the retina and other ocular structures, The anatomical complexity and critical functional role of the OphA mean that any disruption to its blood flow can lead to severe visual consequences (3, 4). When an FD covers the OphA, hemodynamic changes may occur, potentially compromising the OphA’s blood flow patency and leading to various ocular complications, such as vision loss, visual field defects, and retinal embolism (5, 6). These complications not only impair the patient’s visual function but also pose challenges in postoperative management and elevate medical costs. Therefore, a thorough investigation into the risks and effects of FD coverage of the OphA during COA treatment, as well as a systematic evaluation of the mechanisms underlying ocular complications, is crucial for optimizing treatment strategies and enhancing patient outcomes.

Currently, although numerous studies have investigated the effectiveness of FD in treating COA, significant gaps remain in understanding the ocular complications it induces and their underlying mechanisms (7–9). This review systematically examines existing research on FD treatment for COA, with a particular focus on analyzing ocular outcomes and the neuro-ophthalmic complications associated with FD coverage of the OphA. Through a comprehensive analysis of existing studies, we aim to provide new insights and perspectives to guide future research, ultimately improving treatment outcomes, reducing postoperative complications, and enhancing overall patient prognosis.

## 2 The anatomy of OphA

The OphA, as the first major branch of the ICA, originates from the superior edge of the cavernous segment of the ICA. It traverses the optic canal into the orbit, supplying blood to the eyeball and its associated structures (10, 11). A comprehensive understanding of the OphA’s anatomical relationships and clinical significance is crucial for elucidating the formation, progression, and optimal treatment strategies for COA.

The OphA’s primary branches include the Central Retinal Artery (CRA), Short Posterior Ciliary Arteries (SPCA), Anterior Ciliary Arteries (ACA), Palpebral Arteries, Supraorbital Artery, and Supratrochlear Artery (11–13). The CRA travels through the optic nerve to the optic disk, serving as the main blood supply for the retina’s inner layers, which is critical for sustaining vision. The SPCA nourish the outer retinal layers and the choroid, ensuring the metabolism and nutrient delivery necessary for outer retinal tissue function. This dual vascular supply system plays a vital role in preserving retinal integrity and ensuring the accurate transmission of visual signals (11, 12). Additionally, other branches, such as the ACA and Palpebral Arteries, supply blood to the extraocular muscles, eyelids, and conjunctiva, supporting ocular movement and tissue health (11, 12). In addition, the Supraorbital and Supratrochlear Arteries further contribute to blood supply in the forehead and eyebrow regions, emphasizing the OphA’s pivotal role in facial and ocular circulation (14).

## 3 Overview of FD treatment for COA

With continuous technological advancements and the accumulation of clinical practice, FD has demonstrated significant efficacy in the treatment of IA (15, 16). Its application in the management of COA has become increasingly notable (17, 18). However, a thorough evaluation of the clinical efficacy and potential risks associated with FD remains essential. A deeper understanding of the mechanisms underlying FD and its current use in treating COA within the ICA is crucial for advancing intracranial aneurysm research.

### 3.1 Working principle and design features of FD

FD is an innovative endovascular device specifically developed for treating complex intracranial aneurysms, functioning by altering hemodynamics to achieve aneurysm occlusion (19). The primary mechanism of FD involves redirecting blood flow, thereby reducing pressure on the aneurysm wall, facilitating thrombosis within the aneurysm sac, and promoting vascular remodeling and healing (20). FD, primarily composed of nitinol—a material well known for its exceptional biocompatibility, superelasticity, and corrosion resistance—is engineered for long-term stability within the body. Nitinol’s high expansibility and recoverability enable FD to navigate through narrow or tortuous blood vessels during delivery, regaining its intended shape upon reaching the target site (21). These properties ensure FD’s flexibility and precision during implantation while minimizing damage to surrounding tissues. FD is typically constructed with a high-density mesh structure composed of hundreds of fine metal wires, enabling precise endovascular applications. This mesh design uniformly covers the neck of the aneurysm while simultaneously providing a new pathway for redirected blood flow. Two critical parameters—pore size (the dimensions of the mesh gaps) and porosity (the percentage of the total surface area occupied by these gaps)—play a pivotal role in determining the degree of blood flow diversion. A lower porosity, achieved through a denser mesh configuration, exerts greater resistance to blood flow, effectively diminishing the velocity and volume of blood entering the aneurysm. This hemodynamic modulation promotes intra-aneurysmal thrombosis, leading to eventual aneurysm occlusion (22). Moreover, the dense mesh structure fosters endothelial cell proliferation across the vessel wall, further enhancing vascular remodeling and stabilizing the therapeutic outcomes (23).

In clinical practice, once the precise location of the aneurysm is identified, the FD is strategically deployed within the affected vessel, spanning the neck of the aneurysm and anchoring securely to the normal vessel wall. By redirecting blood flow away from the aneurysm sac and into the healthy vasculature, the FD effectively achieves aneurysm exclusion while preserving the structural integrity of the surrounding vasculature, thereby significantly reducing the risk of rupture (24). Following the deployment of the FD, the diminished blood flow velocity and reduced turbulence within the aneurysm sac facilitate thrombus formation. Over time, this thrombus serves to isolate the aneurysm sac from systemic circulation. Concurrently, endothelial cells proliferate and migrate along the surface of the FD, promoting vascular remodeling and ultimately resulting in either complete resorption or stabilization of the aneurysm sac (22).

### 3.2 Clinical application and efficacy of FD for COA

Since the 1990s, extensive discussions in neurosurgical and interventional radiology research have focused on the limitations of conventional approaches to treating intracranial aneurysms. A pivotal randomized controlled trial (RCT) published in 2002 provided compelling evidence that endovascular coiling confers significant advantages over traditional surgical clipping, including improved patient quality of life, a reduction in rebleeding rates, and a lower risk of mortality (25). Another study further highlighted that while both surgical clipping and endovascular coiling effectively prevent aneurysm rupture, they are associated with potential risks, including neurological impairment, infection, and aneurysm recurrence (26). As a result, the quest for safer and more effective therapeutic strategies for aneurysm management has become a central focus in vascular and neurosurgical research.

As the 21st century unfolded, the introduction of FD technology into clinical practice marked a transformative milestone, rapidly establishing itself as a central focus of investigation within the realms of neurosurgery and interventional radiology (27). Considering the complex anatomical structure of the OphA and its essential role in ocular perfusion, FD deployment in COA management has garnered significant clinical attention. A 2017 study demonstrated that FD treatment for COA is both safe and effective, underscoring its feasibility in clinical applications (28). The OphA occlusion rate increased markedly, from an initial 71.8% at baseline to 88.9% at the final follow-up. The first follow-up angiography, conducted approximately 3.2 months post-procedure, highlighted this improvement, which was sustained over an average follow-up period of 31.8 months. Notably, postoperative complications were observed in only two patients: one case of recurrence and one fatality, both attributable to the discontinuation of antiplatelet therapy. In the same year, a comprehensive meta-analysis compared the efficacy of FD, clipping, and coiling in the management of paraclinoid aneurysms. The findings revealed that 71% of patients treated with FD experienced significant visual improvement, surpassing the 58 and 49% improvement rates observed in the clipping and coiling groups, respectively (7). A 2020 meta-analysis further substantiated the efficacy of FD in the treatment of COA, reporting an aneurysm occlusion rate of 85%. Moreover, the incidence of iatrogenic visual complications associated with this intervention was notably low, with new visual symptoms observed in only 3.0% of patients (8). A recent study by Kaiser et al. (29), which included 54 patients with internal carotid artery aneurysms presenting with compressive neuro-ophthalmic symptoms, reported morbidity and mortality rates of 7.4 and 3.7%, respectively. At a median follow-up of 13.3 months, the rates of complete aneurysm occlusion, neck residual, and aneurysm residual were reported as 74, 14, and 12%, respectively. This study effectively demonstrated the efficacy of FD in managing ICA aneurysms associated with compressive neuro-ophthalmic symptoms. Additionally, a meta-analysis conducted by Kaiser et al. (30), encompassing 22 studies and 594 patients, reported recovery, improvement, transient deterioration, and permanent deterioration rates of 47.4, 74.5, 7.1, and 4.9%, respectively, following FD treatment for unruptured ICA aneurysms with compressive neuro-ophthalmic symptoms (NOS). These findings provide further validation of FD’s efficacy in COA management from an ophthalmological standpoint. Collectively, these reports highlight the significant therapeutic benefits of FD in COA treatment and its relatively low complication rate. However, the associated risks warrant careful consideration.

### 3.3 Risks and general complications of FD for COA

Through a systematic review of multiple studies, the use of FD in COA treatment has become increasingly widespread. However, the associated risks and potential complications remain a significant concern in clinical practice.

Ischemic and hemorrhagic complications represent critical postoperative challenges following FD treatment of aneurysms. A study by Brinjikji et al. (31) on ischemic complications demonstrated that while the Pipeline Embolization Device (PED) is generally effective in achieving aneurysm occlusion and reducing recurrence rates, approximately 4.5% of patients developed acute ischemic stroke. The study identified contributing factors such as pre-existing conditions, intraoperative vascular injury, specific aneurysm location and size, and postoperative thrombosis. Furthermore, a comparative study examining ischemic complication rates across various endovascular treatments revealed a significantly higher incidence of ischemic events among patients treated with FD compared to other modalities (32). Moreover, a long-term cohort study (33) reported that 18% of the 413 patients experienced at least one neurological complication, predominantly ischemic in nature and occurring during the early postoperative period. Hemorrhagic complications, occurring in approximately 3.9% of patients, were more frequently observed in cases involving large or complex aneurysms, underscoring the importance of meticulous risk assessment and management.

A comprehensive literature review on hemorrhagic complications related to FD treatment for ICA aneurysms highlights significant risks. Nearly 50% of delayed ruptures are reported to originate from giant aneurysms. Additionally, delayed intraparenchymal hemorrhages (DIPH) represent a severe complication, with an incidence of approximately 2–3%. When considering both delayed rupture and DIPH, it is evident that 70–80% of affected patients experience poor clinical outcomes or mortality, highlighting the critical need for meticulous patient selection and close postoperative monitoring to mitigate these adverse events (34). Due to its metallic composition, FD requires the routine use of dual antiplatelet therapy (DAPT) to reduce thromboembolic risks. However, this therapeutic approach inherently increases the risk of severe bleeding complications, requiring a delicate balance between efficacy and safety in clinical practice (35).

The mechanism and structural characteristics of FD inherently present risks, as its implantation may lead to nerve compression due to device-related factors, inflammatory responses, or thrombosis (22). Improper placement or migration of FD may result in incomplete aneurysm occlusion or vascular injury, emphasizing the critical importance of selecting an appropriately sized device and ensuring precise placement to mitigate these complications (36). A study involving 459 patients revealed that 69 individuals (15.0%) developed in-stent stenosis (ISS), a complication notably prevalent in the Chinese population (37). Furthermore, FD implantation may stimulate the excessive proliferation of vascular endothelial factors, which could contribute to vascular stenosis or occlusion. Additionally, FD implantation may induce excessive proliferation of vascular endothelial factors, potentially leading to vascular stenosis or occlusion (38).

In addition to these complications, long-term follow-up studies have reported rare but severe adverse events, including device fatigue resulting in rupture and vascular perforation (22). Although infrequent, such complications often lead to devastating clinical outcomes. Furthermore, the prolonged administration of antiplatelet therapy during treatment has been associated with adverse drug reactions in certain patients, such as gastrointestinal bleeding and allergic responses (39). While FD offers an effective therapeutic approach for managing aneurysms, its associated risks and complications warrant meticulous evaluation by clinicians. A comprehensive understanding of these potential risks, coupled with timely clinical interventions and diligent long-term management, is essential for optimizing treatment outcomes and improving patient prognosis.

### 3.4 Ophthalmic complications arising from FD coverage of the OphA

Beyond the commonly observed complications, ocular issues following FD treatment of ICA warrant particular attention. The high metal coverage rate and dense mesh design of FD raise concerns about its potential impact on the blood flow patency of the covered OphA, posing risks to ocular health and functionality (5, 6). Studies have demonstrated that, even in cases where the FD covers the OphA and patients exhibit no overt clinical symptoms, objective evidence of OphA occlusion may still be present (5, 40). In clinical practice, some patients treated with FD report ocular abnormalities, including diminished vision, scotomas, and amaurosis fugax, during follow-up examinations. Interestingly, subsequent digital subtraction angiography (DSA) often confirms that the OphA remains patent. A 2015 study systematically evaluated ophthalmic complications in patients undergoing FD treatment for COA and observed a new ophthalmic complication incidence rate as high as 39.3%. These complications predominantly manifested as transient amaurosis fugax, visual field defects, and retinal embolisms (6). A subsequent study published in 2018 reported that up to 40% of patients developed ocular complications following flow diverter (FD) treatment, with 3 cases (18.8%) presenting with amaurosis fugax and 4 cases (25%) experiencing significant visual field defects (38). Additionally, a case report highlighted delayed visual field defects after FD treatment for a paraclinoid aneurysm, underscoring the importance of monitoring for potential postoperative visual impairments (41). More recently, a dual-center study involving 113 aneurysm patients reported that 19% developed varying degrees of ocular complications postoperatively. Although the overall incidence was relatively low and most symptoms were transient, these findings emphasize the need for continued clinical vigilance (1).

In addition to these reports, numerous studies have investigated the ocular complications associated with FD treatment. Some have suggested that OphA occlusion may be influenced by factors such as the coverage area of the device, the duration of coverage, and individual patient-specific differences. Additionally, ischemic ocular symptoms may arise even when the OphA remains patent, likely due to hemodynamic alterations (36, 42). Notably, visual impairments related to FD treatment may not manifest immediately but can develop gradually over months or even years post-treatment. These findings highlight the critical importance of long-term follow-up for the early identification and management of such complications (1).

Some researchers advocate for comprehensive ophthalmic evaluations prior to FD treatment and regular follow-up assessments thereafter to facilitate the early detection and management of ocular complications (6). Diagnostic tools such as visual acuity tests, visual field examinations, and retinal assessments can provide a more precise evaluation of OphA blood flow and its impact on visual function. Furthermore, utilizing Doppler ultrasound to analyze subtle changes in OphA blood flow before and after FD placement, and correlating these findings with ocular complications, may offer more clinically meaningful insights compared to angiographic studies.

Although FD has demonstrated substantial efficacy in managing COA, the potential for ocular complications warrants heightened clinical vigilance. A thorough understanding of the underlying mechanisms and clinical manifestations of ocular complications following FD treatment for ICA is pivotal for optimizing therapeutic outcomes and enhancing patients’ quality of life.

## 4 Ophthalmic outcomes following FD coverage of the OphA

Through a rigorous search and selection process, eight studies were identified that examined ocular outcomes following FD coverage of the OphA during COA treatment. Collectively, these studies analyzed 484 aneurysms in 473 patients who were asymptomatic preoperatively but developed new ocular symptoms following FD coverage. The findings from these studies were systematically reviewed and are comprehensively summarized in Table 1.

**Table 1:** Summary of the baseline characteristics and new visual complications included in the study.

Author	Year	Patients (aneurysms)	Age (years)	Female (%)	Type of FD	Mean max diameter (mm)	OA occlusion rate (%)	New visual complication			Follow-up
								Transient	Persistent	Overall Incidence	
Touzé et al. (38)	2018	15 (16)	48.5 ± 6.1	14 (93.3%)	PED: 68.8%	7.6 ± 4.2	Immediately after procedure: 5 slow-flows and 2 occluded	Amaurosis fugax on the side of the stent: 3 (18.8%)	Unilateral visual field defects: 4 (26.67%)	6 (40%)	4.14 ± 2.03 (year)
					Silk: 12.5%						
					NEG: 12.5%		Last follow-up: 3 occluded (18.8%)				
					FRED: 6.2%						
Rouchaud et al. (6)	2015	28 (28)	52 [19–81]	19 (67.9%)	PED	8 [3–17]	Immediately after procedure: 4 (14.3%)	6 (21.4%)	Retinal emboli: 5 (17.9%)	11 (39.3%)	12 (month)
										Visual blurring: 6	
							3 months: 1 (3.6%)			visual field defects: 4	
							12 months: 1 (3.6%)			Oculomotor palsy: 1	
Bhogal et al. (45)	2017	140 (147)	56.2 ± 13.7	109 (78.1%)	P64. PED	NR	Slow-flows: 11 (8.3%)	1 case (0.71%) experienced visual symptoms after stopping aspirin, but the symptoms disappeared immediately after re taking the medication.			22.3 (month)
							Occluded: 7 (5.3%)				
Puffer et al. (5)	2012	19 (20)	53.9 [23–74]	19 (100%)	PED	NR	Slow-flows: 2 (11%)	Minor entopic phenomena: 1(5.3%)			8.6 ± 3.6 (month)
							Occluded: 4 (21%)				
Durst et al. (46)	2015	19 (19)	53.2 ± 3.07	16 (84%)	PED	10.5 ± 1.43	Slow-flows: 5 (26%)	1 case (5.3%) of blurry vision of the ipsilateral eye after reducing aspirin dosage			12 (month)
Burrows et al. (43)	2016	44 (46)	52 ± 14	41 (93%)	PED: 97.8% Surpass: 2.2%	NR	Occluded: 8 (21.6%)	Peripheral vision loss: 1 (2.2%)	0	3 (6.5%)	29 ± 22 (month)
								2 cases (4.35%) experienced amaurosis fugax that resolved after reestablishing DAPT.			
Levy et al. (1)	2023	113 (113)	59.5 ± 12.4	103 (91.2%)	PED: 6.3%	7.2 ± 3.8	NR	Impaired visual acuity or blurriness: 5 (4.4%)	Permanent visual morbidity: 1 (0.9%)	16 (14.16%)	44.9 (month)
					PED Flex: 51.6%						
					PED Shield: 38.9%			Diplopia: 3 (2.7%)			
					FRED: 2.4%			Field defect: 1 (0.9%)			
					FRED-X: 0.8%			Floaters: 6 (5.3%)			
Chalouhi et al. (44)	2015	95 (95)	53 ± 13	85 (89.5%)	PED	8.0 ± 5.0	Slow-flows: 4 (4.2%)	0	Monocular blindness: 1(1%)	1 (1%)	7.5 [3–24] (month)
							Occluded: 6 (6.3%)				

FD, Flow-diverter; OA, ophthalmic artery; PED, pipeline embolization device; NEG, neuroendograft; FRED, flow redirection endoluminal device; NR, not reported; DAPT, dual antiplatelet therapy.

The analysis of these eight studies highlights substantial variability in the reported ocular outcomes associated with FD coverage of the OphA. Among them, three studies (5, 38, 43) documented an OphA occlusion rate of 20% following FD coverage in a total of 78 patients. In contrast, four other studies (1, 6, 44, 45) reported a markedly lower occlusion rate of approximately 6.6%, based on data from 376 patients. Additionally, a 2020 Meta-Analysis encompassing 16 studies and 913 cases estimated an average OphA occlusion rate of about 10% (8). The overall incidence of ocular complications after FD coverage of the OphA also showed significant variation across the eight studies, ranging from 0 to 40%. For instance, studies by Rouchaud et al. (6) and Touzé et al. (38) reported a total incidence of postoperative ocular complications nearing 40%, whereas the average incidence in the remaining five studies (1, 5, 43–45) was only 5.1%. The same meta-analysis noted an overall incidence of new ophthalmic complications of approximately 3.0% following FD treatment (8). Likewise, the proportion of slowed blood flow in the OphA, as reported in several studies, also varied significantly, ranging from 4.2 to 26% (5, 28, 38, 44, 46).

The observed variations may stem from differences in research methodologies and diagnostic approaches. In the two studies (6, 38) reporting higher incidence rates, researchers conducted specialized and detailed postoperative ophthalmological examinations. For instance, Touzé et al. (38) observed that the mean visual field deviation on the FD-treated side was significantly greater than that on the unaffected side (−1.58 ± 1.12 dB vs. −0.67 ± 1.16 dB, *p* = 0.003). Similarly, Rouchaud et al. (6) emphasized that certain complications could only be identified through comprehensive and meticulous ophthalmological evaluations—assessments that were not routinely performed in most prior studies—further supporting this hypothesis.

The remaining five studies (1, 5, 43–45) predominantly relied on subjective patient-reported outcomes without incorporating professional ophthalmological evaluations. This reliance on subjective perceptions is likely a key factor contributing to the notable discrepancies in reported results. Furthermore, the limitation of small sample sizes in these studies cannot be overlooked. Future research involving larger cohorts is imperative to validate these observations more robustly. Collectively, these findings underscore substantial gaps in the current understanding of ocular complications following FD coverage of the OphA. Addressing these gaps will require more rigorous methodologies and improved diagnostic approaches in subsequent investigations.

## 5 Mechanisms of ophthalmic complications

We conducted a systematic review of the clinical manifestations and existing literature on ocular complications associated with FD treatment. However, an in-depth investigation into the underlying mechanisms contributing to these complications is equally crucial. Recent advancements in FD treatment research suggest that ocular complications may arise from a multifactorial interplay of complex mechanisms. Drawing upon current knowledge, we propose three primary hypothetical mechanisms.

### 5.1 The hypothesis of hemodynamic changes in the OphA

The FD is highly effective in the treatment of aneurysms, and its therapeutic mechanism is intricately linked to the hemodynamics of aneurysms. A comprehensive understanding of the mechanisms underlying aneurysm formation, progression, and rupture is essential to elucidate the role of FD in blood flow redirection. This understanding not only highlights the efficacy of FD but also provides critical insights into the fundamental mechanisms underlying FD-associated complications.

Aneurysms are prevalent cerebrovascular conditions, and their formation, progression, and rupture have been longstanding focal points of research in neurosurgery. Recent studies have increasingly highlighted the pivotal role of hemodynamic parameters in these pathological processes. A 2023 study (47) investigated the relationship between Aneurysm Wall Enhancement (AWE) regions and hemodynamic parameters, demonstrating a significant correlation between aneurysm formation and elevated Wall Shear Stress (WSS) as well as Wall Shear Stress Gradient. Specifically, low Wall Shear Stress (WSS) and elevated Oscillatory Shear Index (OSI) have been implicated in promoting aneurysm formation and progression by inducing pathological vascular remodeling. Another study (48) demonstrated a significant association between low Wall Shear Stress (WSS) and an increased risk of intracranial aneurysm rupture, underscoring the pivotal role of hemodynamics in aneurysm pathophysiology. Specifically, high WSS has been shown to induce destructive remodeling of the arterial wall through sustained mechanical pressure, facilitating aneurysm formation and growth. Conversely, low WSS is linked to flow stagnation and hypoxic regions, which can weaken the arterial wall and elevate rupture risk. The interplay between high and low WSS generates a complex hemodynamic environment that is integral to the formation, progression, and eventual rupture of aneurysms (49).

Structures like the retina and optic nerve are highly metabolically active and demand a substantial supply of oxygen and nutrients. Any insufficiency or hemodynamic alteration can swiftly compromise their function, leading to significant and often rapid impairments in visual performance (50, 51). A recent study utilizing Optical Coherence Tomography Angiography (OCTA) investigated alterations in ocular blood flow parameters before and after retinal disease treatment. The findings revealed significant changes in specific blood flow metrics in the treated eye when compared to the unaffected eye, underscoring the clinical utility of OCTA in monitoring vascular changes associated with retinal interventions (52). This study provides direct evidence of the influence of hemodynamics on ocular function. FD achieves aneurysm treatment by creating a hemodynamic barrier that reduces blood flow into the aneurysm sac. However, when this barrier also extends over the origin of the OphA, it may significantly alter the hemodynamic profile of the OphA, potentially impacting ocular function. A study (53) reported that among 68 covered side branches, 13 (19.1%) exhibited slow blood flow, with 38% of these branches subsequently developing stenosis or occlusion, compared to only 14.5% when blood flow remained stable. These findings suggest that slow blood flow serves as a strong predictor of late side branch occlusion. Consequently, the redirection of blood flow by the FD may alter local oxygen levels, potentially impairing ocular function. This mechanism could underlie the sporadic occurrences of visual decline, transient vision loss, and visual disturbances, such as the appearance of black spots, following FD treatment. Another study (54) investigating the anatomical responses of the choroid and retina following ocular aneurysm surgery lends further support to this perspective. This study was the first to report an increase in the Choroidal Vascular Index (CVI) after FD placement, suggesting that the reduced blood flow in the OphA may trigger angiogenesis on the affected side. These findings further substantiate the hypothesis that FD placement can indirectly influence ocular function by altering local oxygen levels.

Therefore, we hypothesize that the direct influence of FD coverage on OphA blood flow may serve as a critical factor contributing to the elevated incidence of ocular complications observed during the early postoperative stages following FD placement. A recent study (55) revealed that Magnetic Resonance Perfusion (MRP) times were significantly prolonged on the side of FD placement compared to the contralateral side, highlighting the substantial impact of FD coverage on blood flow perfusion in the target vessel and its branches. Another study further corroborates the hypothesis that hypoperfusion may underlie the visual field defects observed on the FD-treated side (38). These findings underscore the critical importance of selecting the appropriate deployment site and device size, not only to ensure the safe and effective occlusion of the aneurysm but also mitigating the risk of ocular complications stemming from insufficient perfusion in the early postoperative stages (36, 42).

In addition to directly altering blood flow dynamics, the role of FD-induced microthrombosis warrants significant attention. The OphA and its branches constitute a sophisticated dual blood supply system for the retina. FD coverage, by altering local hemodynamics, has the potential to precipitate retinal thrombosis, thereby impacting retinal perfusion and function. A study conducted by Rouchaud et al. (6) observed that when the OphA originated from the aneurysm sac, the blood flow pattern transitioned from laminar to turbulent as it passed through the aneurysm. Although the number of such cases was limited, 80% of them exhibited small retinal emboli, which subsequently led to retinal infarction. Another study (1) highlighted that microthrombi migration could obstruct smaller retinal branches, resulting in transient visual disturbances. Building on this observation, Rouchaud et al. (6) further investigated this phenomenon, proposing that high-pressure gradients in covered side branches may help maintain OphA patency. In contrast, lower pressure gradients, such as those found in the OphA, could predispose side branches to thrombosis and occlusion. This mechanism underscores the significant role of FD-induced hemodynamic changes in influencing ocular function.

In the long term, processes such as gradual endothelialization, persistent hypoxia, and prolonged alterations in blood flow dynamics following FD placement may contribute to vascular remodeling and chronic changes, potentially leading to a cascade of complications that compromise the blood supply to ocular structures (56). Following FD placement, neointimal hyperplasia is initiated, facilitating thrombus deposition and triggering an inflammatory response that may stimulate the proliferation of smooth muscle cells. This process can result in significant neointimal overgrowth and vascular stenosis, commonly observed in later stages of stent-induced side branch occlusion (57–60). Studies by Dai and Wang consistently reported varying degrees of neointimal coverage (61, 62), while other research has suggested that neointimal formation postoperatively may be directly associated with complications (63). Conversely, a separate study (44) indicated that elderly patients exhibited higher OphA patency rates, potentially reflecting a diminished neointimal response to stent placement in this population. This indirectly supports the hypothesis that neointimal responses play a crucial role in maintaining OphA patency.

### 5.2 The hypothesis of delayed blood flow compensation

We have previously outlined the anatomical structure of the OphA. Under normal physiological conditions, the eye is predominantly supplied by the ICA. However, when ICA blood flow is restricted or obstructed, the ECA can serve as an alternative source of blood supply through its collateral branches. Studies have demonstrated that in the early stages following FD placement over the OphA, hemodynamic alterations caused by blood flow redirection may transiently reduce the blood supply to the OphA (8). Although the ECA can partially compensate via its branches, this process involves a temporal delay. In scenarios where the collateral flow from the ECA is insufficient and the FD extensively covers the OphA, patients may experience temporary vision loss, transient visual disturbances, or other ocular discomfort due to inadequate blood supply. Over time, however, the compensatory capacity of ECA branches gradually improves, facilitating the establishment of a robust collateral network. This network eventually compensates fully for the ICA supply, effectively maintaining adequate blood flow to the OphA (7, 38, 64).

The study by Rangel-Castilla et al. (40) highlights that despite FD covering up to 90% of the penetrating vessel openings, the reduction in blood flow post-placement is less than 10%. This phenomenon is likely attributable to the presence of collateral networks linking the OphA and ECA branches. In cases where FD placement reduces blood flow, the distal collateral network compensates by supplying terminal organs. Similarly, another study (43) reported that all patients with OphA occlusion eventually regained blood supply via collateral networks, predominantly sourced from ECA branches. Furthermore, research by Zanaty et al. (65) revealed that although approximately 20% of patients experienced OphA occlusion, adequate collateral supply rendered the occlusion clinically insignificant. These findings provide robust evidence for the compensatory capabilities of the ECA. Across most of the included studies (1, 5, 6, 38, 43–45), the majority of newly observed ocular symptoms were transient, which we attribute to the time delay required for the establishment of effective ECA collateral compensation. Once this compensation is fully developed, associated ocular complications resolve and do not recur.

### 5.3 The hypothesis of DAPT

Given the metallic properties of FD, postoperative DAPT is essential to mitigate the risk of thromboembolism. Over the past decade, DAPT has become a standard clinical practice, with aspirin and clopidogrel being the most commonly prescribed antiplatelet agents (66). A multicenter survey conducted in the United States reported that the most prevalent preoperative medication regimen included daily aspirin (325 mg) and clopidogrel (75 mg) for 5 to 7 days. The postoperative maintenance protocol typically comprised daily aspirin (325 mg) for life and clopidogrel (75 mg) for 3 months (67). The latest guidelines recommend that, following Pipeline for the Intracranial Treatment of Aneurysms (PITA) procedures, a daily dose of 100 mg aspirin combined with 75 mg clopidogrel for at least 1 month. Subsequently, patients should continue with a daily aspirin regimen of 100 mg for no less than 6 months. For Pipeline for Uncoilable or Failed Aneurysms (PUFS) treatments, it is advised to administer 325 mg of aspirin daily for a minimum of 6 months, alongside 75 mg of clopidogrel daily for at least 3 months (66).

With the advancement of DAPT research, studies have increasingly highlighted its critical connection to postoperative complications and patient prognosis. A recent meta-analysis revealed that improper use of antiplatelet medications frequently results in symptomatic thrombotic events, including in-stent thrombosis or branch occlusion/stenosis (68). These findings suggest that ocular complications observed after FD coverage of the OphA may also be associated with the non-standard administration of DAPT. For instance, a 2016 study (46), reported a case where a patient, after completing a three-month regimen of clopidogrel, experienced new-onset blurry vision on the ipsilateral side following an unauthorized reduction of the aspirin dose from 325 mg to 81 mg. This change subsequently led to distal focal infarctions in the anterior and middle cerebral artery distributions on the same side.

Reports have documented two cases of acute in-stent thrombosis following FD surgery, both attributed to non-adherence to prescribed antiplatelet regimens. In one instance, complete recanalization was achieved after subacute occlusion of the parent artery, while the other resulted in total occlusion of the internal carotid artery (69). Similarly, another study reported one fatality and one case of permanent morbidity, both linked to the discontinuation of antiplatelet therapy (28). In contrast, a study by Burrows et al. (43) described two cases of suspected transient vision loss occurring at 3 and 6 months postoperatively. Notably, these symptoms resolved entirely upon resumption of dual antiplatelet therapy. These observations further corroborate our hypothesis and underscore the pivotal role of postoperative DAPT in mitigating complications. Ensuring the standardized and consistent use of DAPT in FD device management is essential for minimizing postoperative complications and optimizing patient outcomes.

## 6 Implications and future research

In summary, FD has demonstrated significant efficacy and a relatively low incidence of complications in the treatment of COA. However, despite the substantial advancements these treatment methods have made in clinical practice, postoperative ocular complications remain a critical area of ongoing research. Through a comprehensive review of multiple studies, this paper identifies several key areas that warrant further investigation. First, a common limitation in existing studies is the absence of comprehensive and systematic ophthalmic examinations, coupled with insufficient sample sizes. While some studies have preliminarily explored the relationship between OphA occlusion rates and the incidence of ocular complications, systematic research in this area remains limited. Future studies should focus on expanding sample sizes and incorporating more rigorous, standardized ophthalmic examination techniques to thoroughly investigate the impact of FD coverage on the OphA. Second, changes in OphA hemodynamics during postoperative follow-up represent a critical, yet often overlooked, factor. Hemodynamic alterations can significantly influence the patency of the OphA and ocular health. As discussed earlier, we observed substantial variations in blood flow slowing across the studies included in our review (5, 28, 38, 44, 46). Moreover, it appears that slowed blood flow does not have a direct correlation with postoperative occlusion of the ophthalmic artery or the incidence of postoperative complications. In one of the studies included in our analysis, which involved 19 patients, only 5 patients experienced slowed blood flow postoperatively, yet no ocular complications were reported (46). This may indicate the need for further investigation into the impact of slowed blood flow on ocular outcomes. However, it is important to note that conclusions on this matter have not been consistent across other studies. For instance, the study by Puffer et al. (5) suggested that immediate postoperative blood flow patterns may not serve as reliable predictors of long-term OphA patency, whereas another study (53) indicated that slowed blood flow could be a significant predictor of late side branch occlusion. These conflicting findings highlight the need for further investigation into the postoperative hemodynamic changes in the OphA and their long-term implications for ocular health.

Furthermore, in the majority of the studies included in our review, only the timing of aneurysm and ophthalmic artery occlusion events was documented, while the specific onset of ocular complications was not systematically recorded. This limitation hampers our ability to precisely determine the temporal progression of these complications and impedes a more comprehensive exploration of their underlying mechanisms. Therefore, future research should prioritize the detailed documentation of the timing of ocular complications and incorporate rigorous statistical analyses to facilitate a more nuanced understanding of their temporal dynamics and associated risk factors.

**Figure 2:**
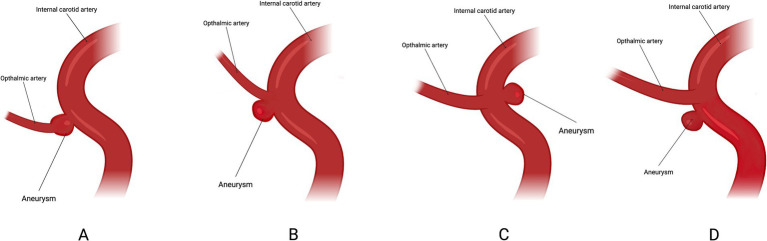
Classification based on the location of the ICA in relation to the ophthalmic artery. Type **A**: ophthalmic artery originating from the aneurysm sac. Type **B**: ophthalmic artery originating from the neck of the aneurysm. Type **C**: ophthalmic artery originating in the inner curve of the carotid siphon. Type **D**: ophthalmic artery not involved in the aneurysm but covered by the flow-diverter.

Most current research on ocular complications associated with FD treatment for COA fails to adequately address the anatomical relationship between the aneurysm and the OphA. However, from a hemodynamic and FD device placement perspective, this relationship is of critical importance. A study (6) categorized the anatomical relationship between the aneurysm and the OphA into four types: Type A, where the OphA originates from within the aneurysm sac; Type B, where the OphA originates from the aneurysm neck; Type C, where the OphA originates from the intradural portion of the ICA; and Type D, where the OphA is unaffected by the aneurysm but is covered by the FD device (Figure 2). The study revealed that, through comprehensive ophthalmic examinations, 5 patients (17.9%) experienced retinal embolism, with 4 of these cases falling under Type A. Moreover, the incidence of ophthalmic complications in Types A and C was significantly higher than in other types. This finding suggests that the anatomical configuration of the OphA is closely related to ophthalmic outcomes. However, due to the small sample size, the ability to detect significant differences between the types was limited. Future studies should focus on further elucidating the relationship between aneurysm anatomy, the OphA, and ocular complications, with an emphasis on expanding sample sizes to provide robust and high-quality evidence.

In addition to anatomical structure, the size of the aneurysm also directly influences the treatment process and outcomes. However, despite the fact that most studies mention the average size of aneurysms, few have explored the specific relationship between aneurysm size and the incidence of ocular complications. For instance, in the study by Rouchaud et al. (6), although aneurysm size and the number of FD devices used were statistically analyzed, the relationship between these factors and ocular complications was not clearly defined. Similarly, in the study by Chalouhi et al. (44), aneurysm size (such as whether it exceeds 7 mm) was identified as an important predictor of OphA patency, with the number of FD and coverage also being key factors. However, these factors were not further investigated in relation to the incidence of complications. This highlights a significant gap in the current research in this field.

The standardized application of dual antiplatelet therapy (DAPT) is essential for minimizing complications and improving prognosis following FD treatment. Currently, most DAPT regimens are based on empirical combination therapies, without individualized protocols specifically tailored to FD treatment (66, 67, 70). Future research should prioritize the development of personalized DAPT regimens for patients undergoing FD treatment of ophthalmic segment aneurysms. Key areas of focus should include determining optimal drug dosages and the appropriate duration of DAPT therapy. In particular, it is essential to investigate whether prolonging the duration of DAPT therapy can effectively reduce the incidence of long-term ocular complications. Such investigations will play a critical role in refining postoperative management strategies, ultimately enhancing treatment outcomes and improving long-term patient prognosis.

Additionally, studies investigating the material properties of FD have highlighted the potential benefits of stents coated with proteins and cytokines possessing distinct antithrombotic properties. These modifications can enhance endothelial layer formation on the stent, contributing to improved outcomes (71). Therefore, the development of specialized stents designed specifically for COA or FD, incorporating tailored surface polymers, may help mitigate OphA occlusion and improve overall treatment efficacy. Moreover, different FD brands, generations (especially coating differences), or sizes (including the number of wires) may have varying effects on the incidence of ocular complications. However, existing literature only provides statistics on the quantity and models of FD used, and lacks systematic answers or detailed comparisons regarding such issues. This gap in research reflects the limitations of current studies and highlights the need for further high-quality research to assess how these factors influence the incidence of ocular complications, thereby providing more reliable evidence for clinical decision-making.

Regrettably, despite providing a relatively comprehensive overview of the mechanisms underlying ocular complications following FD treatment, there remains a significant gap in our understanding of the molecular biological mechanisms that lead to these complications. This indicates that a deeper exploration into the pathogenic molecular mechanisms is essential. Such an investigation may require more focused research from a molecular biology standpoint, rather than solely relying on clinical research perspectives to analyze the pathogenesis.

In conclusion, although extensive research has shown that the use of FD in the treatment of COA generally offers a favorable safety profile with respect to visual outcomes and maintains a relatively low incidence of adverse clinical events, the complication rates remain considerable. These complications continue to significantly impact patients’ quality of life and contribute to an increased economic burden. While current studies have addressed these concerns to some extent, significant gaps in understanding remain. Future research should involve larger-scale, methodologically rigorous investigations to better elucidate the effects of FD on the ophthalmic artery (OphA) and its associated ocular outcomes. Such studies are essential to mitigate postoperative complications and optimize clinical outcomes.
